# Walking Speed Influences the Effects of Implicit Visual Feedback Distortion on Modulation of Gait Symmetry

**DOI:** 10.3389/fnhum.2018.00114

**Published:** 2018-03-26

**Authors:** Gabrielle Maestas, Jiyao Hu, Jessica Trevino, Pranathi Chunduru, Seung-Jae Kim, Hyunglae Lee

**Affiliations:** ^1^School of Biological and Health Systems Engineering, Arizona State University, Tempe, AZ, United States; ^2^Biomedical Engineering, California Baptist University, Riverside, Riverside, CA, United States; ^3^School for Engineering of Matter, Transport, and Energy, Arizona State University, Tempe, AZ, United States

**Keywords:** gait rehabilitation, visual feedback distortion, walking speed, step length symmetry, gait adaptation

## Abstract

The use of visual feedback in gait rehabilitation has been suggested to promote recovery of locomotor function by incorporating interactive visual components. Our prior work demonstrated that visual feedback distortion of changes in step length symmetry entails an implicit or unconscious adaptive process in the subjects’ spatial gait patterns. We investigated whether the effect of the implicit visual feedback distortion would persist at three different walking speeds (slow, self-preferred and fast speeds) and how different walking speeds would affect the amount of adaption. In the visual feedback distortion paradigm, visual vertical bars portraying subjects’ step lengths were distorted so that subjects perceived their step lengths to be asymmetric during testing. Measuring the adjustments in step length during the experiment showed that healthy subjects made spontaneous modulations away from actual symmetry in response to the implicit visual distortion, no matter the walking speed. In all walking scenarios, the effects of implicit distortion became more significant at higher distortion levels. In addition, the amount of adaptation induced by the visual distortion was significantly greater during walking at preferred or slow speed than at the fast speed. These findings indicate that although a link exists between supraspinal function through visual system and human locomotion, sensory feedback control for locomotion is speed-dependent. Ultimately, our results support the concept that implicit visual feedback can act as a dominant form of feedback in gait modulation, regardless of speed.

## Introduction

Gait training is widely used to help individuals with neurological disorders and injuries improve their gait functions (Willoughby et al., 2009; Mehrholz et al., 2014; Schlick et al., 2016). Benefits from treadmill training lie in the coordinated activation of spinal cord central pattern generators from alternating limb movements (Werner et al., 2002; Aagaard, 2003; Dietz, 2003; Eng and Tang, 2007; Plummer et al., 2007). However, clinical results are still not fully satisfactory perhaps because the present forms of gait rehabilitation are not interactive enough to promote an efficient motor learning process (Krebs et al., 2000; Hornby et al., 2008). Treadmill trainings may be improved when accompanied by interactive components because interactive rehabilitation programs keep patients engaged and possibly promote a more efficient adaptive process for motor learning (Schmidt and Young, 1991; Winstein, 1991; Young and Schmidt, 1992).

The use of visual feedback may be one such method of effective interactive training, as it can promote subject participation and its effect can involve an implicit (unconscious or autonomic) adaptive process. Since walking is normally an automatic process, it may be possible that gait adaptation is driven using more of an implicit process than conscious control (Mazzoni and Krakauer, 2006; Bronstein et al., 2009). Thus, implicit processes may play an important role in forming motor adaptation (Reynolds and Bronstein, 2003), and it is of interest to investigate methods of gait training that offer implicit learning. In earlier studies on visual feedback, Kim and colleague (Kim and Krebs, 2012; Kim and Mugisha, 2014; Kim et al., 2015) presented a novel method, called visual feedback distortion, in which the left and right step lengths were measured during treadmill walking and displayed to subjects as two vertical bars on a computer screen. Then, the length of only one bar was distorted without the subjects’ knowledge while they walked at their preferred speed. Even with a distraction task added, subjects spontaneously modulated their gait symmetric pattern away from actual symmetry in response to the distortion. This suggests that the effects of visual feedback distortion in changing step symmetry involve an unconscious adaptive process.

This study extends our earlier studies (Kim and Krebs, 2012; Kim and Mugisha, 2014; Kim et al., 2015) by further investigating whether or not the implicit adaptation, driven by visual feedback distortion, would persist at different walking speeds (slow, preferred and fast speeds) and how different walking speeds affected the amount of adaptation. There is evidence that using visual feedback with treadmills promotes gait modulations (Kim and Krebs, 2012). Extending research on its effectiveness in multiple scenarios with different walking speeds will help researchers to create more efficient therapies by enabling us to predict the long-term effects of using such techniques for therapeutic purposes.

We hypothesized that visual distortion would be effective not only for the preferred walking speed (PWS), but also for fast and slow walking speeds because the effects induced by visual feedback distortion are predominantly mediated through supraspinal function (Kim and Krebs, 2012). Walking speed itself affects visual control, and it has been proposed that either highly automated central pattern generators or the passive tuning mechanism of body dynamics influence gait control more at high speeds, while the active control of high-level sensory feedback, such as vision, appears to be more involved in slower locomotion (Brandt et al., 1999; Jahn et al., 2001; Wuehr et al., 2013). Thus, we also hypothesized that visual feedback distortion may have greater impact on implicit gait adaptation at a slower walking speed and lesser impact at a higher walking speed. Considering that a patient’s pace is usually slow, a positive outcome would suggest potential therapeutic use for implicit visual feedback distortion. This technique may make a positive impact as a gait rehabilitation intervention, if used in conjunction with rehabilitations that produce mechanical perturbations, such as therapies using the instrumented split-belt treadmill (Reisman et al., 2007) or physically interactive robots (Ahn et al., 2011; Ahn and Hogan, 2012; Cajigas et al., 2017).

## Materials and Methods

### Subjects

Twenty-eight healthy, young adult volunteers (17 male, 11 female) with normal or corrected-to-normal vision participated in the experiment. All subjects were familiarized with the treadmill’s function and emergency shut-off capabilities, and provided informed, written consent prior to participating. This study was approved by the Institutional Review Board of Arizona State University. Subjects were told that they would be walking on a treadmill at different speeds and provided with feedback regarding their step length, however they were not told that the feedback would be distorted.

### Experimental Setup

**Figure 1:**
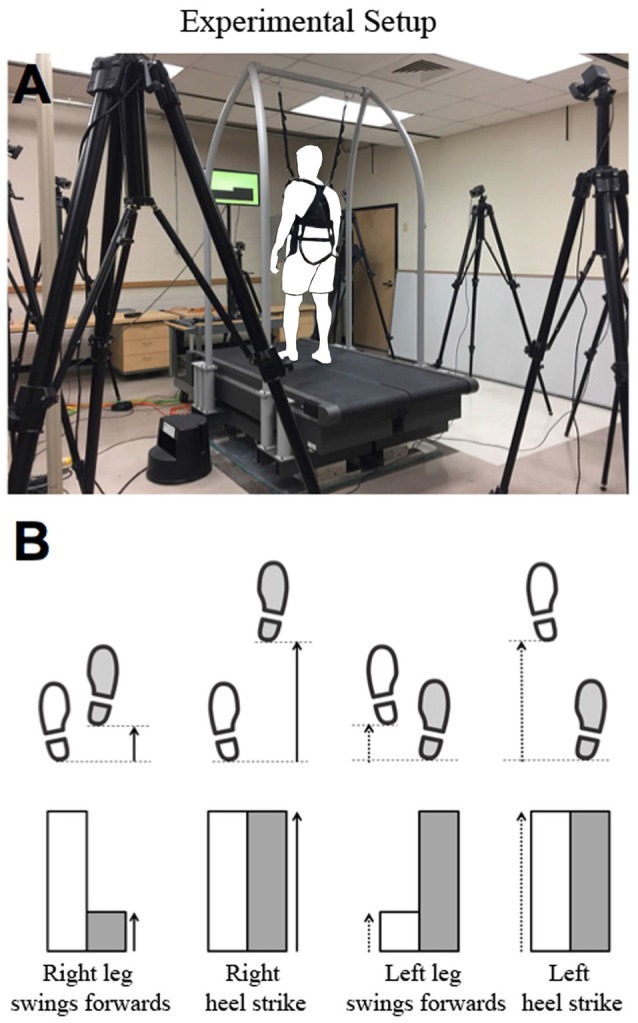
**(A)** Experimental Setup. An instrumented treadmill was accompanied by a safety handle and harness. Eight infrared cameras were placed around the surrounding perimeter to capture the coordinates of 16 markers attached to the subjects’ lower extremities. The size of the bar was calculated in a way that the maximum length would be no greater than around 90% of the monitor screen vertically. **(B)** External visual feedback display. The magnitudes of the right and left step lengths were measured in real-time and plotted in the form of a bar graph, as shown in the bottom row. The bar increased during the swing phase of each leg as the step length increased, and stopped when heel strike occurred. The range of step length mapped to the visual bar was then gradually distorted throughout the experiment.

All walking tasks were performed on the treadmill (Bertec Treadmill, Columbus, OH, USA). A motion capture system (VICON, United Kingdom) was used to capture subjects’ movements. The system utilized eight infrared cameras that detected 16 markers placed on the subjects’ lower extremities to track motion patterns (Figure 1A).

The visual distortion task incorporated a real-time visual feedback system using Vicon SDK to allow subjects to view information regarding step-length. It was implemented in MATLAB code, and introduced to subjects using a 23-inch LCD monitor placed directly in front of the treadmill. Step length was calculated using the heel marker on each foot, and was defined as the distance from one heel marker to the opposite heel marker in the direction of motion. The data for each foot was recorded continuously throughout swing for an instantaneous representation of step length at any time in the experiment. These step length values were then used to create two bar graphs (one bar for each leg) which indicated step length changes in real-time as the subjects walked on the treadmill (Figure 1B). The bar rose in conjunction with the magnitude of the corresponding foot’s step length. Upon heel strike (defined as the moment where each foot’s step length value reached a local maximum during one stride), the maximum height of the bar remained stagnant on the screen until the opposite foot experienced heel strike. This allowed for subjects to view the step length magnitude of each foot for each step.

### Experimental Protocol

Subjects were initially asked to walk for a 5-min training period to determine their PWS. First, subjects began walking at a low speed on the treadmill, and the speed was increased incrementally until the subject found a comfortable walking pace. The speed then rose to a level higher than the indicated comfortable walking speed, and was lowered in decrements until the subject then indicated a second comfortable speed. These two speeds were averaged for the PWS. Slow walking speeds (SLOW) and fast walking speeds (FAST) were then defined as 80% and 120% of the PWS, respectively.

The main experiments were designed as the combination of three types of sessions: PWS, slow walking speed (SLOW) and fast walking speed (FAST). The order of the sessions was randomized. Each subject participated in one of the following four experiments: PWS+SLOW, SLOW+PWS, PWS+FAST and FAST+PWS. Thus, group data for those who participated in SLOW and FAST include 14 subjects, while data for PWS include 28 subjects. Before the main distortion sessions, each subject participated in a 5-min control session, where the subject walked without visual distortion. Walking speed was selected as the same speed in the first training session.

In the distortion sessions, the first testing session was performed at one predetermined speed, while the second testing session was performed at a different predetermined speed. Following the first testing session that the subject participated in, subjects were then asked to rest for a 15-min break. Each testing session was divided into four parts: 1.5 min with no distortion (100%) at the beginning, 9.75 min of distortion, and 3 min of no distortion (100%) at the end. During visual distortion sessions, the distortion increased from 100% to 114% by increments of 2%, then decreased back to 100% by decrements of 2%. Each distortion level lasted for 45 s. Distortions were applied only to the side corresponding to the right leg. For example, at 104% distortion level, the length of the right bar was distorted in increments of 4% of the actual step-length for that side. Subjects would then perceive their step to be 4% greater than it actually was. Subjects were asked to walk comfortably while looking at the graphical feedback of their step symmetry. No clues were given to the subject that the right bar would be distorted throughout the testing period (implicit condition).

### Data Analysis

Step length symmetry, which is defined as the percentage ratio (%) of the left stride length over the right stride length, was recorded during the experiment and analyzed for this study. A ratio of over 100% meant that the right stride length was shorter than the left one, and vice versa for a ratio of less than 100%.

Means and standard deviations (SD) of step length symmetry were calculated for each distortion level within each speed condition for each subject trial. For group analysis, new means and SDs of all subjects were calculated for each distortion level within each speed condition. Erroneous data or outliers were properly removed. Data was defined erroneous when the step length symmetry was either under 70% or over 130%. These outliers resulted from disruptions in the infrared cameras’ abilities to record the location of any marker during the testing period. In addition, any value of step length symmetry lying outside of the mean ± three times the standard deviation was considered an outlier and removed. For each subject trial, the number of erroneous data or outliers was small, less than 1% of the entire data.

To test our first hypothesis that visual distortion is effective not only for the PWS, but also for fast and slow walking speeds, we performed a repeated measures one-way ANOVA on group of step length symmetry. Mauchly’s test of sphericity was used to formally test the assumption of sphericity. If the assumption was violated, the degrees-of-freedom were adjusted using the Greenhouse-Geisser correction before calculating the *p*-value. We further performed paired *t*-tests to determine at which distortion level the step length symmetry significantly differed from baseline, i.e., the initial 100% period. Separate analysis were performed for each of the two subject groups: the slow group (integration of PWS+SLOW and SLOW+PWS) and the fast group (integration of PWS+FAST and FAST+PWS). Separate analyses were also performed for each speed condition: PWS, SLOW and FAST.

To test our second hypothesis that visual distortion at a slower walking speed has a greater impact on implicit gait adaptation, we compared the amounts of adaptation among the three different walking speeds. To minimize subject-to-subject variability, the amount of adaptation for each individual subject’s data set was calculated by subtracting the means of the step length symmetries in either SLOW or FAST (depending on the trial) from those in PWS for each distortion level: PWS−SLOW and PWS−FAST. Each result of PWS−SLOW and PWS−FAST over the entire distortion time intervals was then compared to zero using *t*-tests. A significant positive value indicated a higher amount of adaption in the PWS than the slow or fast speeds. All statistical analyses were performed using SPSS with a significance level of 0.05.

## Results

Among 28 subjects, four were excluded from analysis because they did not exhibit modulation of step length symmetry under both speed conditions. The remaining 24 subjects, six for each of four experimental conditions (PWS+SLOW, SLOW+PWS, PWS+FAST and FAST+PWS), were used in data analyses. PWSs ranged 0.65–1.15 m/s with a mean (SD) of 0.81 (0.13) m/s.

**Figure 2:**
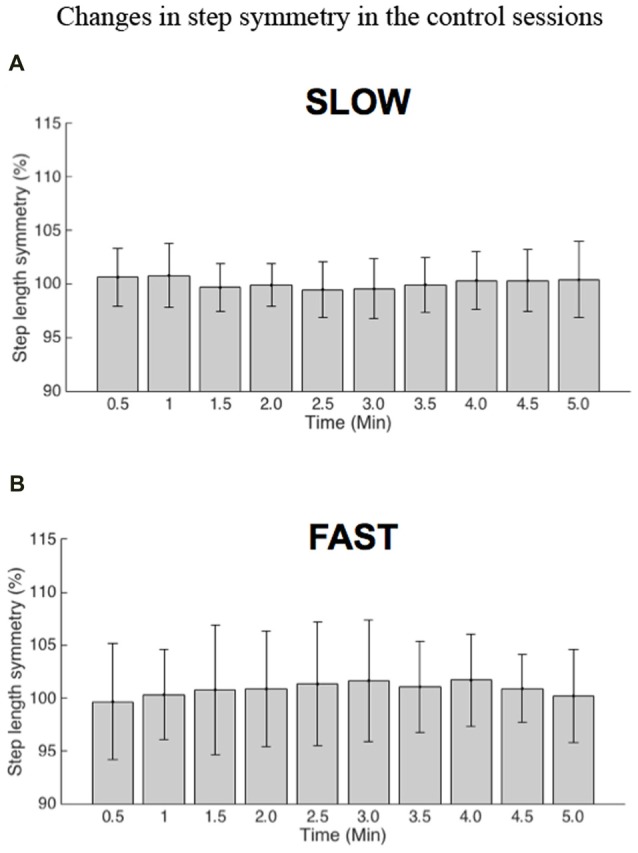
Group results for changes in step length symmetry in the 5-min control sessions without visual distortion. The horizontal axis denotes time in minutes, and the vertical axis shows step length symmetry (the percentage ratio between the left and right step lengths). **(A)** Data from preferred walking speed (PWS)+slow walking speed (SLOW) and SLOW+PWS were integrated in the slow group. **(B)** Data from PWS+fast walking speed (FAST) and FAST+PWS were integrated in the fast group.

During the 5-min control sessions, changes in step length symmetry were negligible. Statistical analysis using repeated measures one-way ANOVA tests confirmed that step length symmetry was statistically not different over time for each 30-s period during the control session: *F*_(2.644,29.089)_ = 0.671, *p* = 0.559 for the SLOW group and *F*_(3.450,37.953)_ = 0.818, *p* = 0.507 for the FAST group (Figure 2).

**Figure 3:**
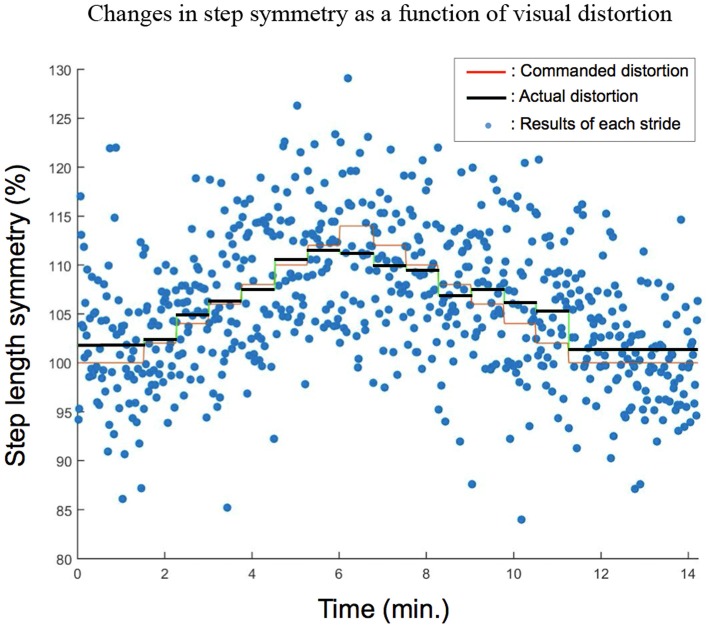
Representative changes in step length symmetry as a function of visual distortion obtained from one sample subject in the PWS condition. The horizontal axis denotes time in minutes, and the vertical axis shows step length symmetry. The orange (faint) line on the graph shows the level of visual distortion that was offered to the subject at each moment. Each dot is indicative of the step length symmetry of the subject at that moment in time. The black (darker) line shows the mean of all dots within each bin.

**Figure 4:**
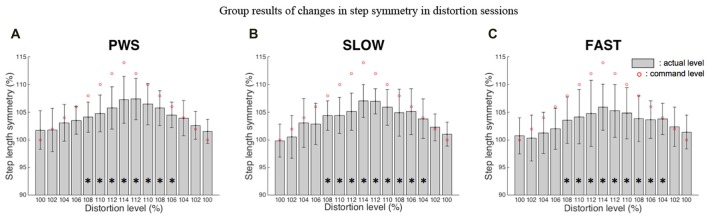
Group changes in step length symmetry in distortion sessions. The bars show step length symmetry averaged across all subjects that participated in the PWS **(A)**, slow walking speed **(B)** and fast walking speed **(C)** trials. Circles indicate the visual distortion applied during the trials. The asterisks (*p < 0.05) mark distortion values where the induced step symmetry values were significantly different from the first 45 s of each trial (having no distortion).

During distortion sessions, it was observed that subjects modulated their step length symmetry away from normal symmetry, regardless of walking speed. An example of changes in step length symmetry as a function of time is shown for one subject in PWS (Figure 3). An upward trend was observed as the level of visual distortion increased, and a downward trend as the level of visual distortion decreased. These trends were consistently observed in all speed conditions shown in group results (Figure 4). Statistical analysis using repeated measures ANOVA tests demonstrated that visual distortion had a significant effect on step length symmetry: *F*_(4.467,98.265)_ = 15.56, *p* < 0.001, *F*_(3.398,33.979)_ = 7.255, *p* < 0.001, and *F*_(4.388,48.271)_ = 9.488, *p* < 0.001 for PWS, SLOW and FAST, respectively. Further analysis using paired *t*-tests showed that the effect became more significant at higher distortion levels as indicated by asterisks (*) in Figure 4. For example, during the period when distortion was increased, significant effects (*p* < 0.05) were found at distortion levels above 106% or 108%.

The amount of adaptation due to visual distortion was significantly greater during walking at the preferred or slow speed than at the fast speed. The difference in gait step symmetry between PWS and SLOW was not statistically different from zero (*t*_(14)_ = −0.215, *p* = 0.83). In contrast, the difference in symmetry between PWS and FAST was positive at all distortion levels (Table 1), and was significantly different from zero (*t*_(14)_ = 9.69, *p* < 0.001).

**Table 1:** Comparison of the amount of adaptation for different walking speeds.

Distortion level	PWS−SLOW	PWS−FAST
100	0.68	2.28
102	0.27	2.42
104	−0.51	2.67
106	0.57	1.62
108	−0.97	1.30
110	−0.11	1.12
112	0.84	0.83
114	−0.31	1.90
112	0.26	2.27
110	0.27	1.93
108	0.69	2.05
106	−0.68	0.95
104	−1.10	1.30
102	−0.29	0.88
100	−0.12	0.72
Mean (SD)	−0.03 (0.61)	1.61 (0.65)

*The differences in gait step symmetry between PWS and SLOW (PWS−SLOW) and between PWS and FAST (PWS−FAST) were calculated and compared to zero. PWS−SLOW: *p* > 0.05, PWS−FAST: *p* ≪ 0.001*.

## Discussion

In this study, we have investigated the effects of implicit visual feedback distortion on gait modulation at multiple walking speeds. We studied speed effects and dose responses when subjects were presented with distorted visual feedback.

### Adaptation of Gait Symmetry in Response to Visual Feedback Distortion

The results demonstrate that subjects spontaneously modulated their gait symmetric pattern in response to implicit visual feedback distortion, regardless of speed conditions. This indicates that subjects can be manipulated away from their typical sensory feedback loops when provided with outside feedback that is assumed to be objective. The observed gait modulations may be evidence that supraspinal function is dominant during visual feedback, even over other proprioceptive signals from lower limbs. However, not all participants reacted to the visual distortion; 4 out of 28 subjects showed neither upward nor downward trends in step symmetry as the distortion level changed, and their symmetries looked similar to those of their control trials. Although we cannot rule out a neurophysical explanation, these subjects might not have concentrated on the viewing screen during their trials.

The visual feedback used in this study did not generate any visual perception of motion. Instead, it simply displayed information about subjects’ step lengths as two vertical bars. Subjects were instructed to walk comfortably while looking at the vertical bars during the experiment, and no other instructions were given. During trials, they were unaware of the distortion of the bar graph. All subjects even reported, during post-experiment debriefing, that they did not realize the visual distortion or recognize changes in their gait patterns. Nevertheless, subjects unintentionally changed their gait symmetry when one of the bars representing step length was distorted, which is corroborated by the results from our previous study (Kim and Krebs, 2012). Our speculation is that the self-walking motion comes from not only physical senses, but also from our innate psychological notion of walking symmetry (Butt et al., 2002; Ivanenko et al., 2009) perhaps because bipedal humans have been walking in such a fashion for most of their lives. If observation through external visual feedback appears to differ from that predicted knowledge, gait adaptation would occur in order to compensate for the discrepancy. Our findings support previous work on visuomotor adaptation of arm movements (Tseng et al., 2007; Schaefer et al., 2012), although the adaptive mechanisms with respect to leg and arm movements may be quite different. Prior studies on visuomotor adaptation of arm reaching suggested that the motor system cannot tolerate differences between the trajectory planned through a prediction of motor commands (the so-called forward model) and the executed trajectory perceived in a visual space through visual feedback (Kawato, 1999; Mazzoni and Krakauer, 2006). While such sensory prediction errors occur, the motor system may involve implicit strategy more than explicit strategy because such changes are generally not seen as intended by subjects (Bronstein et al., 2009). Adaptation to distorted visual feedback occurs through sensorimotor adaptation and also other forms of learning that are thought to be less implicit and influenced by instructions and the use of feedback (Morehead et al., 2017). In other words, any source of feedback that signals task success or failure can drive changes in behavior, but these changes are different from the adaptation of an internal model from sensory prediction errors (Miyamoto et al., 2014). In our study, because no task was given to the subjects, the feedback was task-irrelevant. However, we cannot rule out the possibility that the subjects consciously corrected the movement during the trial of distorted visual feedback. The use of implicit visual feedback distortion involves sensory prediction error-based learning that is involuntary (Kim and Mugisha, 2014), but gait adaptation observed in this study may reflect the composite effects of task performance and sensory prediction errors.

### Effects of Walking Speed on Gait Adaptation

Our results showed that the subjects’ gait modulation happened at all three walking speeds (Figure 4). Our statistical analysis showed no overall significant difference in gait step symmetry at all distortion levels between the slow and PWSs. However, the amount of adaptation induced for the fast speed was significantly smaller than for the preferred speed. These results indicate that visual distortion had a more significant effect on changes in step length symmetry at the slow and preferred speeds than the fast speed. This may indicate that the faster gait and slower gait are controlled by somewhat different actions of the gait control. Wuehr et al. suggested that gait control involves a combination of processes that are fine-tuned by the spontaneous interaction of supraspinal neural control, multisensory feedback control, automated central pattern generators, and passive biomechanical tuning mechanisms of body dynamics (Wuehr et al., 2013). Increased walking speed may have a stabilizing influence on subjects’ balance employing more of an automatic spinal program of gait control and/or the passive tuning mechanism (Brandt et al., 1999). Once the highly automatic gait control has been initiated, it may possibly suppress the effect of visual distortion on gait symmetric modulation. In contrast, at the slow speed, the engagement of sensory prediction error-based learning may be relatively increased, making the subjects more malleable to gait modulation in response to visual feedback distortion.

### Utilizing Visual Feedback Distortion as a Potential Rehabilitation Intervention

Gait rehabilitation often incorporates interactive visual components to optimize treadmill training. Using visual feedback can engage the individual in clinical training, promoting motor learning. Not limited to this, vision has also been shown to have an influence on adjustments in walking speed and stride length (Konczak, 1994; Prokop et al., 1997). It has additionally been suggested that visual perturbations delivered at different phases of the gait cycle can result in altered leg motions (Logan et al., 2014). Taking this into account, the use of visual feedback for future rehabilitation may be very promising. We have presented real-time feedback of step length symmetry in a simple format using bar graphs. As a result, subjects spontaneously modulated their step length symmetry at all walking speeds in response to implicit visual feedback distortion, and the effects of distortion were more significant at higher distortion levels for all scenarios. In addition, we observed some differences in the slope of induced step symmetry between the increasing and the decreasing distortion periods (Figure 4). This is consistent with our previous study (Kim and Krebs, 2012) and may indicate the quick adaptation of the subjects to the implicit visual feedback distortion. By integrating this same visual feedback distortion into gait rehabilitation, we can then understand whether this influence upon step symmetry remains true for those whose motor control and interpretation of visual feedback may suffer as a result of age or disease. We envision promising therapeutic interventions in which visual feedback distortion applied during walking may improve the efficacy of gait training. Further work is needed to understand whether these changes in symmetry occur in patient populations.

## Conclusion

This study investigated how an implicit visual distortion affects gait symmetry across different walking speeds, where simple bar graphs representing subjects’ right and left step length were presented. The results showed that subjects made spontaneous modulations to their gait symmetries in response to the visual feedback distortion at all walking speeds. In addition, the amount of induced modulation was significantly greater during walking at the preferred or slow speed than at the fast speed. This suggests that the visual distortion method has a great effect on implicit gait modulation, and this effect is related to walking speed.

## Author Contributions

HL and S-JK conceived and planned the experiments and analyzed the data. GM, JH, JT and PC carried out the experiments. GM, HL and S-JK wrote the manuscript with input from all authors.

## Conflict of Interest Statement

The authors declare that the research was conducted in the absence of any commercial or financial relationships that could be construed as a potential conflict of interest.
